# Effect of magnesium sulfate in oxidized lipid bilayers properties by using molecular dynamics

**DOI:** 10.1016/j.bbrep.2021.100998

**Published:** 2021-04-28

**Authors:** Miguel Fernández, Reinaldo Marín, Fulgencio Proverbio, Fernando Ruette

**Affiliations:** a Laboratorio de Química Computacional, Centro de Química, Instituto Venezolano de Investigaciones Científicas (IVIC), Apartado Postal 21827, Caracas, 1020A, Venezuela; b Laboratorio de Bioenergética Celular, Centro de Biofísica y Bioquímica, Instituto Venezolano de Investigaciones Científicas (IVIC), IVIC-CBB, Apartado Postal 21827, Caracas, 1020A, Venezuela

**Keywords:** Lipid peroxidation, Magnesium sulfate, Membrane modeling, Molecular dynamics, Oxidized membrane, Magnesium protection

## Abstract

Magnesium sulfate (MgSO_4_) has been used as a protector agent for many diseases related to oxidative stress. The effect of MgSO_4_ on the oxidized lipid bilayer has not yet been studied using molecular dynamics calculations. In this work, the effects of oxidation were evaluated by using a POPC membrane model at different concentrations of its aldehyde (-CHO) and hydroperoxide (-OOH) derivatives with and without MgSO_4_. Several quantitative and qualitative properties were evaluated, such as membrane thickness, area per lipid, area compressibility modulus, snapshots after simulation finish, density distributions, time evolutions of oxidized group positions, and radial distributions of oxidized group concerning Mg. Results indicate that in the absence of MgSO_4_ the mobility of oxidized groups, particularly –CHO, toward the surface interface is high. At a low oxidation level of the bilayer there is an increase in the compressibility modulus as compared to the unoxidized bilayer. MgSO_4_, at a low oxidation level, tends to lessen the oxidation effects by lowering the dispersion in the distribution of oxidized species toward the membrane surface and the water region. However, MgSO_4_ does not change the trends of decreasing membrane thickness and area compressibility modulus and increasing area per lipid upon oxidation. In this regard, MgSO_4_ diminishes the electrostatic long-distance attractive interactions between the oxidized groups and the charged headgroups of the interface, owing to the Mg^+2^ and SO_4_^-2^ screening effects and an electrostatic stabilization of the headgroups, preventing the pore formation, which is well-known to occur in oxidized membranes.

## Highlights

• MgSO_4_ in vitro restores oxidized membranes but its molecular mechanism is unclear.
• MD simulations of oxidized lipid bilayers were performed with and without of MgSO_4_.
• A restriction in the mobility of oxidized groups is produced by MgSO_4_.
• Mg^+2^ and SO_4_^=^ produce screening effects on the oxidized membranes.
• MgSO_4_ produce a diminution of electrostatic long-distance attractive interactions.

## 1 Introduction

All cells are surrounded by a plasma membrane that separates their interior from the extracellular environment. It is widely accepted that the cell membrane is a complex structure that functions to protect the cell and its organelles, as well as to control the influx and efflux of various substances through the intrinsic permeability of the lipid bilayer, the activity of channels, and transporters located in the membrane. A lipid bilayer, such as a plasma membrane, can be affected by a lipid peroxidation (LP) process which is well-known to play an important role in cell membrane damage because alters its physiological functions. This process starts with the action of reactive oxygen species (ROS), normally generated as a result of intracellular metabolism that may function as signaling molecules [1]. Excessive ROS production can be triggered by several internal inflammatory processes as well as external agents, such as UV light or environmental toxic compounds [2]. The increase of ROS production together with a decrease in the antioxidant defense mechanisms could produce an imbalance that leads to oxidative stress, which could damage proteins, lipids, and DNA, disrupting in this way in numerous diseases.

The LP is initiated by a mechanism of free radical (e.g., ^●^OH, ^1^O_2_) chain reaction, leading to the formation of lipid hydroperoxides (ROOH) in the fatty acid residues of the lipid bilayer [3]. In most cases, it affects polyunsaturated aliphatic chains because of their contents of double bonds separated by methylene groups (–CH_2_–), which have particularly reactive hydrogen atoms. Besides, hydroperoxide species, truncated chains with aldehyde or carboxylic groups are typically products of membrane oxidation. The incorporation of these oxide groups in the unsaturated chains dramatically alters the phospholipid amphipathic character [4].

The injury caused to cell membranes by oxidative stress has been associated with several diseases and inflammatory responses in humans and animals [5], such as Parkinson's [6,7], Alzheimer's [[7], [8], [9]], hypoxia/reoxygenation [[10], [11], [12], [13], [14], [15]], preeclampsia [[16], [17], [18], [19], [20], [21], [22], [23], [24]], hypertension [19,25], renal failure [25,26], pulmonary illness [27], cancer [28,29], and other pathological processes [30].

Molecular modeling, using molecular dynamics (MD) methods have been extensively employed to elucidate properties of cell membranes [[31], [32], [41], [33], [34], [35], [36], [37], [38], [39], [40]]. In particular, there are many theoretical studies [[42], [43], [52], [53], [54], [55], [56], [57], [58], [59], [60], [61], [44], [62], [63], [64], [65], [66], [67], [68], [69], [45], [46], [47], [48], [49], [50], [51]] of oxidized membranes using MD with atomistic force fields and others complemented with experimental Results [4,55,58,68,[70], [71], [72], [73], [74]]. These studies seek to elucidate the effect of oxidized lipids on the molecular structure of lipid bilayers for several reactive oxygen species, lipid peroxidation, and antioxidative defense mechanisms [3,30,75,76].

A typical example of membrane property changes due to LP is given by Wong-ekkabut et al. work [42]. These authors evaluated the effects of replacing normal lipids with their oxidized forms (aldehydes and hydroperoxides) in palmitoyl-linoleyl-phosphatidylcholine bilayers (PLPC), ranging from 0 to 50% concentrations. They found major structural changes on oxidized lipids because the polar tails bend to the interface with the water due to the formation of hydrogen bonds between the water molecules and the polar head groups. The augmented concentration of oxidized lipids increases the area per lipid, decreases the membrane thickness, reduces the order parameter of the aliphatic chains, and rises the permeability of the water through the membrane.

In the literature, magnesium salts have been experimentally reported to decrease the LP effects, e.g. in preeclampsia [[16], [17], [18], [19], [20], [21], [22], [23], [24]]. Thus, Chiarello et al. [20] concluded that the treatment of MgSO⁠_4_ in women with preeclampsia has been beneficial in the restoring of endothelial functions, inactivating brain N-methyl-d-aspartate (NMDA) receptors, reducing the inflammatory response, and the oxidative stress; being not only beneficial to the mother but also the neonate.

Magnesium is an element related to many physiological pathways (cofactor for more than 300 enzymes) that include: energy production, synthesis of essential molecules, structural roles, ion transport across cell membranes, cell signaling, cell migration, and nutrient interactions [77,78]. All of them are related to functions, such as muscle contraction, neuromuscular conduction, glycemic control, myocardial contraction, and blood pressure.

Several experiments with animals have been performed with magnesium to protect and to prevent neuronal apoptosis due to neonatal hypoxic-ischemic brain injury in pregnant rats [12]. It has been also reported MgSO_4_ as a scavenger of free oxygen radicals to ameliorate perinatal hypoxia/reoxygenation brain damage [13] and the protection of fetal skin from intrauterine ischemia-reperfusion injury [11]. Besides, the prophylactic treatment with MgSO_4_ reduces the observed changes in ischemia/reperfusion injury in the ovary [79]. Furthermore, renal dysfunction and oxidative stress can be lessened by MgSO_4_ administration in streptozotocin-induced diabetic rats [26]. Interestingly, MgSO_4_ supplementation significantly prevented heat stress-induced oxidative damage in broiler chickens. This effect seems to be mediated, at least partly, by restoring heat stress-impaired activities of the anti-oxidative enzymes including superoxide dismutase, catalase and glutathione peroxidase, together with a reversal of the heat stress-induced lipid peroxidation [80].

In humans, Hartwing [81] found that Mg, besides its stabilizing effect on DNA and chromatin structure, is also an essential cofactor for almost all enzymatic systems involved in DNA processing. On the other hand, Goñi de Cerio et al. [82] reviewed the neuroprotective therapies after perinatal hypoxia/reoxygenation brain injury and reported that one of the most useful therapies is based on MgSO_4_. In this sense, Lingam and Robertson [83] made a review on Mg as neuroprotective agents used in the fetus, term infant with neonatal encephalopathy, and adult patients with a stroke. In general, MgSO_4_ plays an important role in clinical healthcare [84] and intensive care units [85]. Also, there are other important applications of magnesium compounds used in the food industry as an antioxidant [86] and cellulose protection [87]. Furthermore, magnesium deficiency in plants is becoming an increasingly severe problem to be solved in the cereal industry because, these days, most people absorb lower Mg than before of starting with the heavy chemical fertilization in agriculture [88].

Besides the diversity of applications to humans, animal experiments, plants, and the industry in general, MgSO_4_ protects and prevents the cell membrane from oxidative damage caused by hydroxyl radicals not only in vivo but also in vitro [20]. Hence, this salt provides antioxidant protection and also stabilizes the structure of oxidized plasma membranes [20]. A possible explanation, regarding the antioxidant protection of MgSO_4_ in vitro and in vivo, is given in previous work by Fernández et al. [89]. The authors performed computational simulation and modeling, by using quantum chemistry and molecular dynamics (MD) calculations for a model bilayer membrane and MgSO_4_ hydrated ion pairs. It was shown that this salt is adsorbed over the membrane surface close to the phosphate groups and besides, it can react with hydroxyl radicals to form ionic pair complexes, where the unpaired-electron radical is stabilized by resonance on the sulfate (SO_⁠4_⁠^−2^) S=O double bonds. In this way, the MgSO_4_ salt traps hydroxyl radicals, preventing further membrane oxidation. Also, the preincubation of oxidized membranes either in vivo or in vitro, with MgSO_4_ can diminish their LP effects. Nevertheless, a theoretical modeling study of MgSO_4_ effects on oxidized membranes has not been performed yet; as far as we know. Therefore, researchers are required to understand, at the molecular level, the in vitro MgSO_4_ effects on oxidized membranes. Note that in vivo, in addition to the intrinsic antioxidant effect of MgSO_4_, there is a magnesium activity in the synthesis of glutathione [90,91], catalase [92] and membrane repairing mechanisms [93,94].

In this study, the effect of MgSO_4_ on an oxidized lipid bilayer model is evaluated using MD with an atomistic force field. Simulations of 1-palmitoyl-2-oleyl-sn-glycero-3-phosphatidylcholine bilayers (POPC) with different concentrations of their oxidized derivatives (-CHO and –OOH) were performed. Subsequently, simulations of these systems in the presence of MgSO_4_ with a fixed concentration were also conducted to analyze the effect of this salt in the membrane structure.

This work is organized as follows: Section 2 describes the models employed for oxidized membranes and the method and the used algorithms for MD calculations, including the evaluated properties and software for visualization and plotting. Results of membrane qualitative calculations for oxidized membrane with and without MgSO_4_, considering the different percentages of oxidation are given in Section 3.1, including comparison with literature calculations of similar systems. Discussion of quantitative property calculations for non-oxidized and oxidized bilayers with and without MgSO_4_ presence is shown in Section 3.2. Comparison between our results in oxidized membrane and literature proposed mechanisms of Mg protection are analyzed in Section 3.3. Finally, in Section 4, the main conclusions and comments are presented for the effect of this salt on model oxidized cell membranes.

## 2 Methods

The lipid bilayer model used in this work is composed of 128 molecules of 1-palmitoyl-2-oleoyl-sn-glycero-3-phosphatidylcholine (POPC), 64 per layer, with 5535 molecules of water in a periodic box with ~6.2 × 6.2 × 8.5 nm (X, Y, and Z lengths, respectively) obtained from previous work [89]. The main groups present, after a membrane has been exposed to LP conditions, are hydroperoxide, aldehyde, and carboxylic acid [61]. In this sense, a lipid bilayer simulation with the hydroperoxide (1-palmitoyl-2-(9-hydroperoxy-cis-octadece-10-noyl)-sn-glycero-3-phosphatidylcholine (PCHP)) and the aldehyde (1-palmitoyl-2-(9-oxononanoyl)-sn-glycero-3-phosphatidylcholine (PCAL)) may be representative of an oxidized cell membrane.

**Fig. 1:**
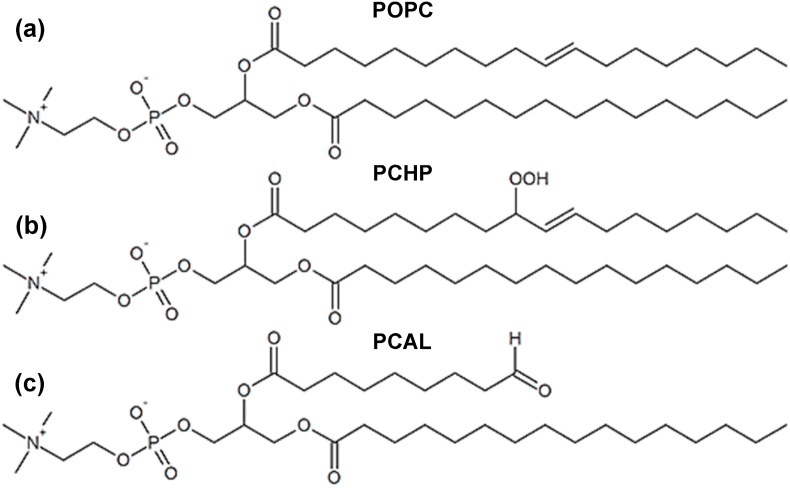
Structures of non-oxidized and oxidized lipid chains: (a) 1-palmitoyl-2-oleoyl-sn-glycero-3-phosphatidylcholine (POPC) (b) 1-palmitoyl-2-(9-hydroperoxy-cis-octadecen-10-oyl)-sn-glycero-3-phosphatidylcholine (PCHP). (c) l-palmitoyl-2-(9-oxo-nonanoyl)-sn-glycero-3-phosphatidylcholine (PCAL).

In this study, 8, 16, and 32 lipid molecules per layer (corresponding to 12.5, 25, and 50% oxidized concentration) were randomly substituted by their oxidized forms of the same type, (PCHP or PCAL). A picture of the unoxidized and oxidized lipids are shown in Fig. 1. In this model, four (4) molecules of MgSO_4_ in the form of contact ion pair was selected as starting point with a structure similar to that proposed in previous papers [89,95]. There is no restriction to the movement of Mg–O(S) interaction. This number of MgSO_4_ molecules corresponds to a concentration of 18.7 mM that is equal to 3.125% concerning the number of lipid molecules. The MgSO_4_ molecules were also randomly added to the final structures of the oxidized systems in the water zone. This small selected concentration is usually administered intravenously with magnesium sulfate to patients with conditions related to oxidative stress [96].

All the calculations were performed with the GROMACS-5.1 [97] program. The configurations and steps of calculations were the same as those used in a previous study [89]. The parameters for simulations were compatible with the all atoms force field of CHARM36 [98]. In this sense, the conditions of simulation for this force field were kept as close as possible to the values published by Piggot et al. [31]. The sulfate parameters were obtained from CgenFF [99], and the Mg parameter values were obtained from the parameterizations described by Allnér et al. [100], who developed the best parameters to reproduce the experimental kinetic data and a good magnesium ion–water coordination simulation. The parameters for PCHP and PCAL of the oxidize section were assigned from those of 5-hydroperoxy-cis-non-3-ene and pentanal by using the Ligand Reader and Modeler module [101] contained in CHARMM-GUI [102].

The hydrogen bonds were constrained using the LINCS algorithm [103]. The neighbor list was updated every 10 steps using a cutoff radius of 1.2 nm. Long-range electrostatic interactions were treated by the particle-mesh Ewald (PME) method [104] with a cutoff radius of 1.2 nm, a grid spacing of 0.12 nm, and cubic interpolation. The switching off function for van der Waals interactions was chosen between 0.8 and 1.2 nm. The time step for all calculations was 2 fs. Initial structures were minimized with the steepest descent algorithm. The simulations were carried out in two steps. Firstly, simulations with the NVT ensemble were performed during 500 ps using the modified-Berendsen thermostat [105] to equilibrate temperature at 298 K. Secondly, simulations with the NPT ensemble were carried out employing the Nosé–Hoover thermostat [106,107] and the Parinello-Rahman barostat [108], at 298 K and 1 bar of pressure. In this case, each NPT simulation was of 1μs, for a total of about 12 μs of simulations, considering oxidized systems PCHP and PCAL at three different concentrations with and without MgSO_4_. All calculations with MgSO_4_ start with the final calculated structure of oxidized species.

For the determination of structural changes in the lipid bilayer, without and with MgSO_4_, snapshots of oxidized lipid bilayer atoms and time evolution of oxidized group positions along the Z-direction for 50% oxidized lipids were depicted. In addition, the density distributions of P atoms and oxidized molecular groups (-CHO and –OOH) and the radial distribution functions (RDF) of these groups referent to Mg were displayed for 12.5, 25, and 50% of oxidized species. Also, the variation of thickness, area per lipid, and area compressibility modulus was evaluated and their variations with the percentage of oxidation were graphed. The VMD [109] and the Grace [110] software were used to visualize the membrane structure and plotting, respectively.

## 3 Results and discussion

As mentioned in the Introduction, LP alters the properties of cell membranes, and MgSO_4_ partially restores these properties on oxidized cell membranes. Therefore, this section is divided into three parts: (a) A comparison of qualitative oxidized lipid bilayer properties considering the different percentages of oxidations in the presence and absence of MgSO_4_ as well as the comparison with previous studies. (b) Discussion of standard calculated oxidized bilayer properties and the effect of the presence of MgSO_4_. (c) An overview of the evaluation of the MgSO_4_ effects over cell membranes exposed to oxidative conditions by a comparison between theoretical with experimental data and an explanation of MgSO_4_ action.

### 3.1 Qualitative effects of oxidized bilayers without and with MgSO_4_

It is well-known that lipid oxidation on cell membranes Results in alterations in their properties [69,74]. The main changes, associated with peroxidation of the membranes, are loss of their characteristic permeability, phase separation of lipids, cross-linking of polar head groups, and the increase of the transbilayer lipid movements. It has been established that these changes are due to alterations in the conformational dynamics of modified aliphatic chains by peroxidation. For this reason, a qualitative location of all species after simulation can give relevant information relative to the alterations of the oxidized cell membranes.

#### 3.1.1 Comparison of snapshots with 50% of oxidation

**Fig. 2:**
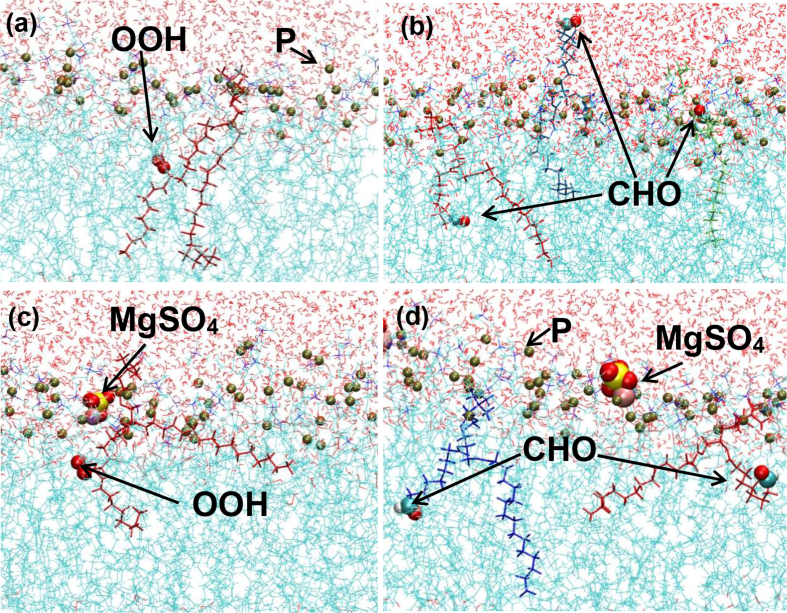
Snapshots of a 50% oxidized lipid bilayer with the –OOH and –CHO groups: (a) for PCHP and (b) for PCAL without MgSO_4_; (c) for PCHP and (d) for PCAL with MgSO_4_. Blue and red wires correspond to phospholipids chains and water molecules, respectively. Contrasting and bold chains are oxidized phospholipids. Gray, red, white, blue, violet, and yellow balls are P, O, H, C, Mg, and S atoms, respectively. (For interpretation of the references to colour in this figure legend, the reader is referred to the Web version of this article).

To visualize the location of oxidized species in the membrane, snapshots of final structures from calculations with 50% of PCHP and with 50% of PCAL are displayed in Fig. 2. A rearrangement of the hydroperoxide groups (-OOH) bent and moved toward the polar headgroups, close to the interface with water, is observed in Fig. 2a. On the other hand, Fig. 2b reveals that the –CHO can be found immersed in the aqueous phase. In this sense, various theoretical studies have shown that the oxidized groups bent toward interface due to their capacity to establish hydrogen bonds with water molecules [42,61,74]. The same trends were also found by several MD calculations [53,59,69,73]. For example, Siani et al. [69] showed Results of MD cross-grain simulations using ELBA and MARTINI force-fields on several types of an oxidized bilayer of membrane models. They conclude that –OOH groups do not induce pore formation independently of the water model and force field employed, contrary to the study of Wong-ekkabut et al. [42]. Siani also reported the tendency of the polar oxidized groups to move toward the water interface, leading to an oxidized lipid tail becoming crooked and resulting in a structural membrane reorganization.

For comparison, Fig. 2 also shows captures of POPC/PCHP and POPC/PCAL systems at 50% oxidized species, in the presence of MgSO_4_. It can be seen that MgSO_4_ is located mainly on the water-membrane interface; i.e., adsorbed on head groups of the bilayer surface. The Mg^+2^ cations are close to the phosphate groups (PO_4_^-^) as expected by electrostatic interactions. Notice that the SO_4_^-2^ groups are also close to the Mg^+2^ cations. In the case of PCHP, the location of –OOH groups (see Fig. 2c) suggest a long-range interaction with the MgSO_4_ close to the interface with water (see discussion below). On the other hand, the –CHO group of PCAL is located close to the interface, but no into the aqueous phase (see Fig. 2d). The comparison between Fig. 2a and b with Fig. 2c and d, respectively, indicates that the presence of MgSO_4_ has an important influence on the location of the oxidized groups, particularly for the PCAL ones. The fact that calculations with MgSO_4_ start with the final calculated structure of oxidized species in the water region implies that this salt reverts the location of –CHO groups.

#### 3.1.2 Comparison of the density distribution

**Fig. 3:**
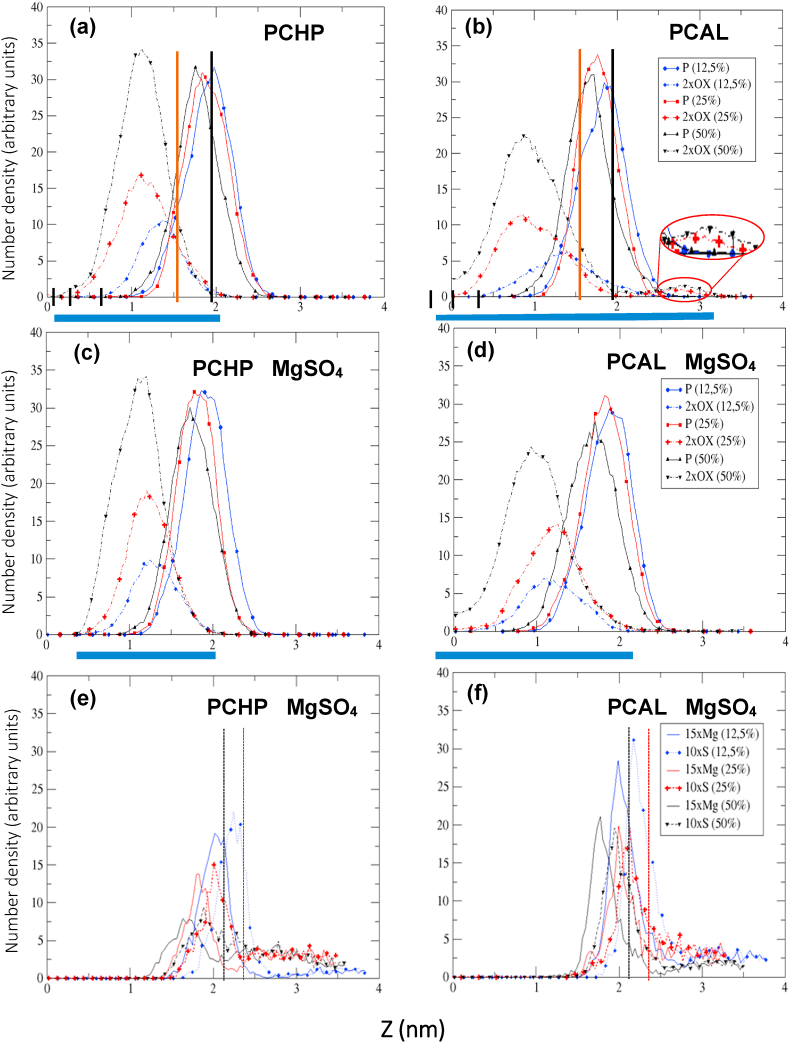
Density distribution (DD) for P, oxidized groups (OX: –OOH or –CHO) and MgSO_4_ groups (Mg^+2^ and S (SO_4_^-2^)) with 12.5, 25 and 50% of oxidized species. Systems without MgSO_4_: (a) P and –OOH, (b) P and –CHO. Systems with MgSO_4_: (c) P and –OOH, (d) P and –COH, (e) Mg and S with PCHP, (f) Mg and S with PCAL. Z = 0 corresponds to the bilayer center. Horizontal blue gross lines show the width of the oxidized group for 50% oxidation. Black and brown vertical lines and black and red dotted vertical lines correspond to the DD peak position of phosphate, ester, Mg and S groups, respectively, in the non-oxidized membrane (see Reference [89]). (For interpretation of the references to colour in this figure legend, the reader is referred to the Web version of this article).

The density distribution (DD) for POPC systems with 12.5, 25, and 50% of oxidized species (PCHP and PCAL) in the presence and absence of MgSO_4_ are displayed in Fig. 3a–d for the P and –OOH and –CHO groups. In Fig. 3e and f, the DD for Mg and S are also depicted for different percentages of oxidized species. The distributions are across the membrane (Z length), in which Z = 0 corresponds to the center of the lipid bilayer (see horizontal axis in Fig. 3) considering only a layer because bilayers, as considered in this study, are symmetric. Results show that the membrane thickness (headgroup-headgroup distance) decreases with the percentage of oxidized species, see the location of phosphate peaks concerning the non-oxidized membrane represented by vertical backlines in Fig. 3a and b. This trend is in agreement with the results reported in the literature [42,59,69,73]. The oxidized groups are mainly located below the ester groups of the lipids referred to as vertical brown lines shown in Fig. 3a and b, but there is an important overlapping with the phosphate group densities. The same trends are shown by Wong-ekkabut et al. [42] in similar systems.

Regarding the distribution of the oxidized groups, the Results reveal a great difference between the –OOH and –CHO groups. For example, the spread of DD for 50% of oxidation, depicted as a horizontal blue gross line below the ordinates in Fig. 3a and b shows a greater dispersion for –CHO than for –OOH group. It is also clear that the dispersion of the oxidized groups increases as the oxidation increases, see small vertical black bars at the left side of the distribution, which are moving close to the middle of the membrane (Z = 0), as the oxidation raises. The distribution of hydroperoxide groups is relatively narrow, which is due to the formation of H-bonds with water molecules, carbonyl and phosphate groups, where these specific short-range interactions tend to favor the location of –OOH groups in the vicinity of polar heads but also transiently dive deeper into the membrane (see Garrec et al. [47]). In the case of the –CHO group (Fig. 3b) the distribution is wider. It can be noticed that the increase of PCAL concentration increases the distribution or spreading of these species, appearing at a small peak in the water zone, as shown in the inset. Also, an increase in the DD value beyond Z = 0 is observed, due to interactions with oxidized species of the other layer. Similar results were reported by Van der Paal et al. [49] with a system composed of POPC and the corresponding aldehyde-oxidized form. These results suggest that the –CHO group exhibits high mobility between different layers and can come into contact with each other as the concentration of oxidized species increases. Notice that, even with the small truncated aldehyde chain, a penetration of the oxidized groups (OX) into the other layer (see density value at Z = 0) is observed at 50% of oxidation. These results are in agreement with the tilt angle distributions of the oxidized lipid tails models reported by Jarerattanachat et al. [46] and Boonoy et al. [48], showing that the distribution for –CHO groups is wider than that for –OOH groups and they can reach contact in the opposing leaflet. In another work, Lee and Malmstadt [61] using also an atomistic MD approach found that at concentrations of 18 mol % or less, the –CHO terminal of a shortened oxidized-lipid tail tends to interact with water and thus bends toward the bilayer-water interface, in agreement with previous experiments and simulations. In particular, these authors proposed that these changes allow water molecules to pass through the oxidized bilayer without pore formation, what they called as the passive permeability of oxidized bilayers.

The density distributions for POPC systems with 12.5, 25, and 50% oxidized species (PCAL and PCHP) with MgSO_4_ are displayed in Fig. 3c and d. It can be seen that there is a reduction of the dispersion of oxidized species by the presence of MgSO_4_ with respect to without it (compare of blue bar lengths below ordinates in Fig. 3a and b with those in Fig. 3c and d, respectively). Notice that CHO group of the oxidized chain is not placed into the water region (see Fig. 3d). This indicates that the MgSO_4_ sensibly affects the motion of the –CHO groups. In this sense, Boonnoy et al. [51] showed that aldehyde mobility is fundamental for the formation of pores in lipid bilayers; then, the observed MgSO_4_ restriction in the aldehyde motion Results in an inhibition of pore formation mechanism and therefore stabilization of the cell membrane structure.

The DD for Mg and S were also calculated as shown in Fig. 3e and f. In general, it can be observed that Mg and S are located close to the membrane surface, where the phosphates and choline are found (see the locations of P in Fig. 3c and d). This finding has been previously reported for the unoxidized membrane [89]. As the membrane is oxidized, the P moves toward the middle of the membrane because the witness decreases. This trend is also observed for Mg and S, see the location of Mg and S for 12.5%, 25%, and 50% with blue, red, and black lines, respectively, with reference to black and red dotted vertical lines for non-oxidized membrane. For systems with PCHP (Fig. 3e), it is observed that, as the concentration of oxidized lipids rises, the maxima of Mg and S decrease and their densities spread increase. This also occurs with PCAL, but in a lesser extent. This indicates that, as oxidation increases, Mg and S are dispersed from the membrane surface. It means that there is an important interaction between the oxidized species and Mg^+2^ and SO_4_^-2^ ions. In the case of PCAL systems (Fig. 3f), the maxima are higher than for PCHP and the changes due to oxidation are smaller compared to the latter. This means the density distributions of Mg and S are more sensitive to PCHP than PCAL.

#### 3.1.3 Comparison of oxidized group time evolution positions

**Fig. 4:**
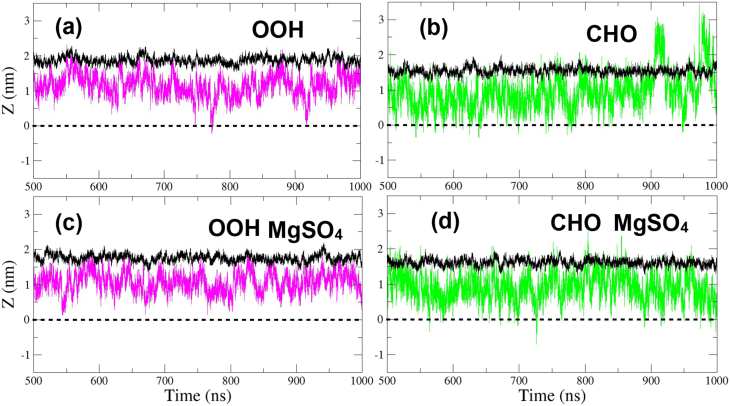
The time evolution of oxidized group positions along the Z-direction for a 50% oxidized lipids mixture. (a) PCHP and (b) PCAL without MgSO_4;_ (c) for PCHP and (d) for PCAL with MgSO_4_. Pink and green lines correspond to –OOH and –CHO groups, respectively. The black line and the doted black one represent the average position of the phosphate group and the Z = 0, respectively. (For interpretation of the references to colour in this figure legend, the reader is referred to the Web version of this article).

Fig. 4 shows the position of a hydroperoxide (pink broken line) and aldehyde groups (green broken line) on the Z-axis as a function of time, for the 50% oxidized species systems concerning the average position of the phosphate group (dark broken line) and the middle of the bilayer (dotted line). It is observed that the –OOH group remains in an approximately selected region close to the phosphates with eventual crossings to the other layer (Fig. 4a). On the other hand, the high mobility of the –CHO group can be noticed (Fig. 4b), confirming the Results of a snapshot at the end of the simulation (Fig. 2b) and the DD graphic (Fig. 3b), showing some PCAL positions out in the water region (above the dark broken line) and also in the other layer (below the dotted line).

The position of oxidized groups on the Z-axis as a function of the time at 50% of oxidized species with MgSO_4_ is given for the system with PCHP in Fig. 4c. This shows that the trajectory of the hydroperoxide group is restricted to be between the phosphate and the middle of the bilayer concerning the system without MgSO_4_ (Fig. 4a). A more drastic change is observed for the –CHO group where it seldom exceeds the average position of the phosphates (Fig. 4d), confirming the fact that MgSO_4_ decreases the mobility of the aldehyde groups (see Fig. 4b for comparison).

#### 3.1.4 Magnesium effect in the radial distribution of oxidized species

**Fig. 5:**
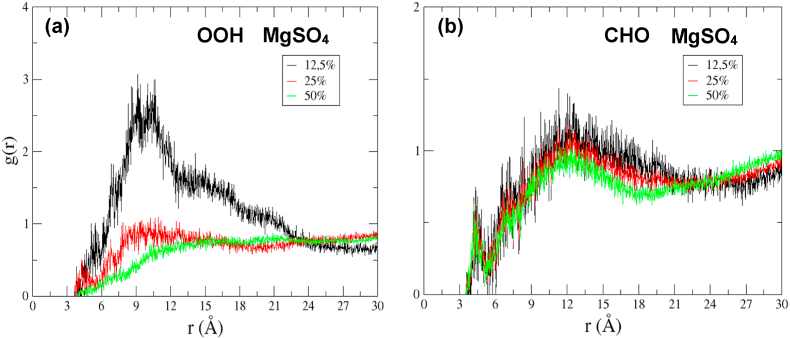
RDFs for O atoms concerning Mg atoms: (a) O of OH from the –OOH group of PCHP system and (b) O of –CHO group of PCAL system with 12.5, 25, and 50% of oxidized species, respectively.

To evaluate possible correlations between the location of Mg and oxidized species, the radial distribution function (RDF) between oxygen atoms of the oxidized group (O linked to H in OOH and O linked to C in CHO), concerning Mg atoms at different concentrations of oxidized species, are displayed in Fig. 5. In the case of PCHP systems, clear peaks of dark lines, around 9 and 11 Å, are observed for the system with 12.5% oxidation. This means that there is a correlation in the Z-axis (in the surface membrane direction) between the place of the PCHP chains and the location of Mg^+2^. For the system with 25% oxidation, the correlation decreases, and a small non-well-defined peak around 9 Å is detected; besides, a small peak at 4.5 Å is observed. In the case of the system with 50% oxidation, the Mg effect on the location of PCHP around 9 Å disappears. On the other hand, in systems with PCAL, a maximum is detected around 12 Å with small changes with the concentration; also, a small peak at 4.5 Å can be observed, for all concentrations.

In general, as an average, two types of interactions may be determined for both systems: one being at long-range (9–11 Å for hydroperoxides and 12 Å for aldehydes) and another of smaller magnitude at short-range (4.5 Å), where the oxygen of the oxidized groups are found close to the second solvation layer of Mg^+2^. The interactions between PCAL and particularly PCHP with Mg^+2^ become less effective as their concentration increases because of the increasing interaction between oxidized species and to a fixed small salt concentration that limits locations of the Mg^+2^ cations on the membrane surface. For a small concentration of PCHP, Mg^+2^ can be located from oxidized groups at around 9 Å, but when the oxidation increases this relationship tends to disappear (see red and green broken lines in Fig. 5a). In the case of PCAL, the long-distance interaction slightly decreases with the concentration of this species, because the lateral interaction between them is lesser than PCHP due to its smaller functional group and shorted chain. Thus, these Results are in agreement with the decrease in the mobility of the aldehyde group as observed in the time evolution along the Z-direction (see Fig. 4d) and a narrower density dispersion in the DD referent to oxidized systems without Mg^+2^ (see Fig. 3c–d). Notice that the magnesium effect is mainly in the diffusion of oxidized species in the Z-direction but not in the lateral diffusion.

### 3.2 Quantitative comparison of calculated properties of non-oxidized and oxidized phospholipids with and without MgSO_4_

**Fig. 6:**
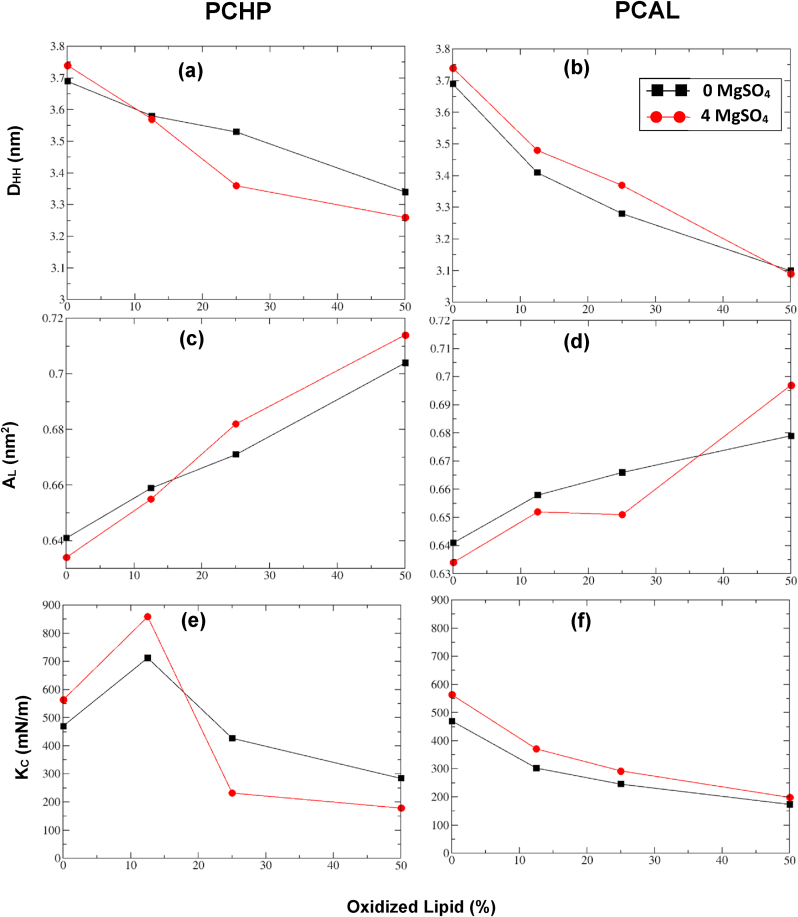
Property changes with the percentage of oxidized species with and without MgSO_4_. (a) and (b) thickness (D_HH_); (c) and (d) area per lipid (A_L_); and (e) and (f) area compressibility modulus (K_C_). The POPC oxidized bilayer lipids (PCHP and PCAL) are ranging from 0% to 50%. The black and red lines and points correspond to the cases without and with MgSO_4_, respectively. (For interpretation of the references to colour in this figure legend, the reader is referred to the Web version of this article.)

In the literature, the mean value of headgroup-headgroup bilayer thickness (D_HH_), area per lipid (A_L_), and area compressibility modulus (K_C_) are the commons parameters calculated for lipid bilayer systems, since they represent the basic structural properties [42,59,69,73]. To evaluate the effects of lipid peroxidation on membranes, calculations of these lipid bilayer properties at a different percentage of oxidation were determined with and without MgSO_4_. The case of 0% membrane oxidation is also included from Reference [89] for comparison, as shown in Table 1 and Fig. 6.

**Table 1:** POPC bilayer properties: average values of headgroup-headgroup bilayer thickness (D_HH_), average area per lipid (A_L_), and area compressibility modulus (K_C_) with the oxidized species at 0.0, 12.5, 25, 50% for PCHP and PCAL in presence of MgSO_4_. Values in parentheses correspond to oxidized systems without MgSO_4_.

Oxidized Lipid Species	Oxidized Lipid Concentration (%)	D_HH_ (nm)	A_L_(nm^2^)	K_C_ (mN/m)
none*	0.0	3.74 ± 0.19	0.635 ± 0.009	564.2 ± 7.6
		(3.69 ± 0.20)	(0.641 ± 0.009)	(470.0 ± 6.9)
PCHP	12.5	3.57 ± 0.20	0.655 ± 0.007	859.0 ± 9.2
		(3.58 ± 0.21)	(0.659 ± 0.008)	(712.4 ± 8.3)
	25	3.36 ± 0.20	0.682 ± 0.014	232.2 ± 4.7
		(3.53 ± 0.20)	(0.671 ± 0.010)	(427.4 ± 6.4)
	50	3.26 ± 0.20	0.714 ± 0.016	178.7 ± 4.0
		(3.34 ± 0.19)	(0.704 ± 0.013)	(284.8 ± 5.1)
PCAL	12.5	3.48 ± 0.21	0.652 ± 0.011	371.3 ± 6.1
		(3.41 ± 0.21)	(0.658 ± 0.012)	(303.1 ± 6.1)
	25	3.37 ± 0.23	0.651 ± 0.012	292.7 ± 5.4
		(3.28 ± 0.18)	(0.666 ± 0.013)	(246.4 ± 4.4)
	50	3.09 ± 0.25	0.697 ± 0.015	199.0 ± 4.3
		(3.10 ± 0.23)	(0.679 ± 0.016)	(173.8 ± 4.1)

*Values from Reference [89].

Results for a bilayer with and without MgSO_4_ clearly show, that as the bilayer is more oxidized, the D_HH_ decreases, and the A_L_ increases (see Fig. 6a–d and values in Table 1). These trends, as mentioned above, are in agreement with the results reported in similar studies [42,59,69,73] for oxidized membranes without Mg^2+^. The K_C_, in general (Fig. 6e–f), decreases, but for PCHP increases at a low oxidation level without and with MgSO_4_. This last issue is in concordance with results observed in Fig. 5a, where there is a strong correlation between the locations of –OOH groups with respect to Mg at 12.5% of oxidation. Experimental results by Suda et al. [111] at low percentages of oxidation, using an artificial cell membrane system and fluorescence recovery technique, found a lower diffusion coefficient for PCHP than in a non-oxidized lipid, while for a truncated lipid (PCAL) a higher diffusion coefficient was reported. This last feature makes that the trimmed fatty acid chains of PCAL do not pack together tightly; therefore, the tails can move freely within the interior of the membrane, and proteins and phospholipids can diffuse laterally through the membrane. In the case of the oxidized PCHP membrane at low concentrations is more difficult the lateral diffusion because of the volume of the –OOH groups. If the tail volume increases because of the presence of –OOH groups the head groups come close together in the interface layer at a low percentage of oxidation concentration and the lateral movement decreases. At a higher percentage (25 and 50%) head groups tend to be more separated and then there is an important increase in A_L_ and the attractive electrostatic interaction becomes weaker to allow lateral diffusion.

Atomistic MD simulations performed by Kumar et al. [59] reported that, upon oxidation, the oxidized group (-OOH) migrates towards the aqueous phase and the backbone of 5α-CH tilts, which causes the membrane to expand laterally. Also, the reactive oxygen species (ROS) can migrate through the membrane because of the decrease of breaching barriers that may lead to apoptosis. Due to the decrease of the thickness and the increase of the area per lipid, they proposed that lipid oxidation increases membrane curvature and permeability. In another type of system, using a lipid bilayer from the mitochondrial membrane of cardiolipin, Vähäheikkilä et al. [53] also found that oxidized groups shift closer to the membrane-water interface region, forming hydrogen bonds with several other groups. They also reported a conformational change that turned out to in a decrease of the bilayer thickness and an increase of the area per lipid. On the other hand, Weber et al. [73], using biomimetic membranes, reported similar induced structural changes by the oxidation of POPC and DOPC phospholipids. They performed experimental research using irradiation to a vesicle decorated with the anchored photosensitizer that generates singlet oxygen species ^1^O_2_ that induce oxidized lipids. A decrease of the membrane stretching modulus (Kc) as a function of the fraction of oxidized lipids was reported as a consequence of a corresponding increase of the lipid area and the subsequent increase of the total area of the vesicle.

The increase of the lipid area leads to a weaker lipid-lipid interaction (less cohesive energy) because of a larger separation between them and also a less distance between interlayer head-head groups (decrease of thickness). This induces a large elasticity and, in general, a decrease of K_C_ constant. These Results are in correlation with experimental studies by Abad et al. [19] where it is observed an increase in the osmotic fragility of red blood cells from normotensive and preeclamptic women with oxidative stress, as a result of changes in form and flexibility of the cell membranes.

The analysis of properties (P) with the degree of oxidation, as shown in Table 1, indicates that aldehyde (PCAL) species lead to a large change in D_HH_ and K_C_ properties concerning the hydroperoxide ones (PCHP). A quantitative way to show the percentage of P changes (ΔP%) is given in Eq. (1).

(1) $$\Delta P\;\%=\frac{\left|\left(P\left(0\;\%\right)-P(50\;\%\right)\right|}{\left|(P\left(0\;\%\right)\right|},$$

where P(0%) is the membrane property value with 0% of oxidation and without MgSO_4_. The application of this equation to the systems without MgSO_4_ gave values of 9.5 and 16.0% for D_HH_, and 39.4and 63.0% for K_C_ to PCHP and PCAL, respectively. The change in thickness for PCHP (9.5%) is in good agreement with Weber's et al. [73] Results of roughly 10% for bilayers with 50% of hydroperoxidized lipids. The stronger change in K_C_ for aldehyde at 50% of oxidation would indicate that there is more tendency to form pores in –CHO than in –OOH lipids, as proposed by Boonoy et al. [48]. On the other hand, in the case of A_L,_ the change percentage gives values of 9.8 and 5.9% for PCHP and PCAL, respectively. The greater value in PCHP than in PCAL may be explained by the trunked PCAL chain produces less hindrance with neighbors than the PCHP and the possibility of being in the water region, reducing the interaction, as shown in the snapshot displayed in Fig. 2b.

The MgSO_4_ has shown the capability to in some way to restore the properties of red cell membranes after been exposed to oxidizing conditions [21]. In this way, calculations of quantitative properties with a certain concentration of oxidized lipid models and low MgSO_4_ concentration will be important to estimate the effects of this salt over an oxidized lipid bilayer. The membrane properties with oxidized species (PCAL and PCHP) with and without MgSO_4_ are presented in Table 1 and Fig. 6. It is observed that the increase of membrane oxidation produces the trend to decrease mean values of D_HH_ and K_C_ and an increase of A_L_. The MgSO_4_ does not prevent it at a high oxidation concentration (50%). In fact, Results of application of Eq. (1) show that ΔP% values, in general, with MgSO_4_ (D_HH_ (11.6 and 16.3%), A_L_ (11.4 and 8.7%), and K_C_ (62.0 and 57.7%)) are greater than without it (D_HH_ (9.5 and 16.0%), A_L_ (9.8 and 5.9%), and K_C_ (39.4 and 63.3%)) for PCHP and PCAL, respectively.

The effect on the degree of change concerning oxidation percentage is, however, different depending on the percentage of oxidized species and the type of property. For example, D_HH_ and K_C_ for PCAL with MgSO_4_ decrease less than without it, at concentrations of 12.5 and 25% (see Fig. 6a and f). Similarly, A_L_ increases less with the presence of MgSO_4_, except for an oxidation percentage of 50% (see Fig. 6d). On the other hand, for PCHP, the MgSO_4_ influence is only observed at values lower or close to 12.5% (see Fig. 6a, c, and 6e).

These Results are not in agreement with the work of Jarerattanachat et al. [46] using NaCl as a salt, for an oxidized lipid bilayer of 1-palmitoyl-2-linoleoyl-sn-glycero-3-phosphatidylcholine (PLPC). They found that the thickness increases and the area per lipid decreases. However, the NaCl salt is completely dissociated where the monocation Na^+^ permeates into the bilayer headgroup region, while the dication Mg^+2^ is located on the interface region close to the phosphate head groups and near to SO_4_^-2^ forming an ion pair in which the immediate vicinity are amino groups. Also, the MgSO_4_ concentration in this work is quite different (about 19 mM) as compared with the NaCl concentrations of those authors (0.1 and 1 M) [46].

### 3.3 Effects of MgSO_4_ on oxidized lipid bilayers

Several experimental studies are dealing with the effects of MgSO_4_ on cell membrane systems under oxidation conditions, indicating that this salt attenuates the damage produced by LP [10,17,21,22,24,79].

Although the MgSO_4_ cell protection may be associated with the fact that this salt supports the cellular mechanisms responsible for restoring cell membranes [94], it is also observed in in vitro systems where this cannot occur, which indicates that direct interaction of this salt with the membrane is associated with its protection. In this way, the Results shown in this work indicate the existence of a prevention mechanism for cell membranes with a certain degree of oxidation mediated by MgSO_4_. Hence, our results reveal that MgSO_4_ decreases the mobility of aldehyde groups in the direction of the membrane surface, which are associated with pore formation in lipid bilayers exposed to oxidative conditions. In fact, experimental results on artificial cell membranes by Tero et al. [112] found that protrusions appeared on the lipid bilayer surface before the formation of nanopores, which may be attributed as nanopore precursors. So, MgSO_4_ can lessen the possibility that the oxidized groups (-CHO) move to the hydrophilic region to form protrusions that could lead to the pore formation.

It is important to mention that MgSO_4_, in general, does not diminish the changes in membrane properties (D_HH_, A_L_, K_C_) at different degrees of oxidation. A clear comparison is displayed in a graph of bilayer property changes with the degree of oxidation, with and without the salt (see Fig. 6). This indicates that there is a limit to the recovery effect of the salt, depending on the oxidant group and the oxidation concentration. The effect of this salt is more effective for low membrane oxidation. In general, the presence of Mg^+2^ attenuates the oxidation effects (a decrease of D_HH_, an increase of A_L_, and a decrease of K_C_) for the oxidized bilayer, especially at reduced oxidation. It is important to notice that levels of oxidation in the cell membranes, associated with various pathologies, are normally low [63]; therefore, in this work, the selected Mg^+2^ concentration was about 19 mM. It is also relevant to emphasize that the main influence of Mg^+2^ on the oxidized bilayer is to maintain its stability related to the mobility or dispersion of the oxidized group in the Z direction, even in the highest oxidized case (50% of oxidation).

Another important issue to point out is that hydroperoxides should not be the majority of oxidized groups in a membrane, because they are relatively unstable, giving secondary products of lipid peroxidation, such as aldehydes [113]. These are the main groups in the oxidized membrane that create great damage because highly increase membrane permeability, phase segregation, and flip-flop movement of lipids, leading to pore formation [5].

Several interpretations of the MgSO_4_ effect in experimental observations suggest that this salt is directly related to the structural stability of cell membranes. Thus, Tomov et al. [114] showed that MgSO_4_ prevents hemolysis of erythrocytes in vitro exposed to electrical pulses in a medium of low ionic strength. These authors propose that Mg^+2^ reduces the electrostatic repulsion and stabilizes the negatively charged groups of lipids and proteins in the membrane. Similarly, Tongyai et al. [115], suggested that Mg^+2^ strengthens the erythrocyte membrane and increases its electrical stability, which induces a decrease in the number and size of the erythrocyte pores. On the other hand, Dupuy-Fons et al. [116] reported that Mg salts can reverse heating-induced red blood cell stiffening without modifying the flexibility of the membranes. Also, Martín-Molina et al. [117] by using experimental techniques (phase analysis light scattering, to measure electrophoretic mobility) and MD showed that Mg^+2^ (MgNO_3_), contrary to Ca^+2^ (CaNO_3_), is involved in binding of two lipids through their phosphate or carboxylic moieties. They proposed two different locations: one more superficial and one more profound. Hence, Mg^+2^, in addition to restricting the mobility of the phosphate groups, restores the stability in oxidized cell membranes.

Other work by Bara et al. [118] presents a model to explain the magnesium salt effects on the stabilization or destabilization of amniotic cell membranes based on electrostatic interactions and ionic bonds. These authors suggested a screening effect, which is based on an indirect long-distance interaction of the solvated ions with the charged groups of the lipid bilayers. They concluded that membrane stability is a unique action at low Mg concentration, increasing their electrical resistance because divalent cations are located near the surface of the membrane and close to negative surface sites.

In this work and the previous one [89], the Mg^+2^ cations are located after simulations at the interface water membrane close to the negative phosphate and sulfate charges, which also are near amino sites on the interface. This produces an increase in the interaction between two or more head groups, which is also related to the stabilization of the oxidized membranes. Furthermore, the decrease of electrostatic long-distance interaction due to Mg^+2^ and SO_4_^-2^ shieldings are responsible for the decrease of the oxidized group interaction with the charged interface lipid ions, lessening oxidative effects on the membrane. This is experimentally reported in the red cell osmotic fragility and the restoration of the activity of a membrane protein, such as the Ca-ATPase, which plays an important role in the fine control of the intracellular concentration of Ca^+2^ [21].

Finally, as a result of this work, previous one, and the review of experimental Results, a general explanation of the MgSO_4_ activity in the LP on cell membranes is proposed: (a) This salt would contribute to the antioxidant processes of cells by decreasing the ^●^OH reactivity due to direct interaction with Mg^+2^ and a spin delocalization on the sulfate ligand, avoiding continuous oxidation of the membrane [89]. (b) Magnesium inhibits the membrane disruption by preventing the pore formation due to a decrease of oxidized lipid mobility toward the membrane surface [this work]. (c) It is known that Mg^+2^ is a cofactor that participates in the synthesis of several cell antioxidants [92]. (d) This salt promotes the formation of enzymes for the natural repair of damaged membranes with pore formation due to lipid oxidation [94].

## 4 Concluding remarks

MgSO_4_ plays an important role in the antioxidant defense, specifically against lipid peroxidation. It can inhibit the ^●^OH activity for LP and has the potentiality of reestablishing some membrane properties that lessen in oxidized cell membranes. However, despite the intense experimental research and the great importance for human health, the molecular mechanisms of action associated with this salt in the oxidized membrane have not been established yet. In this sense, through computational simulations in a previous study [89], it was shown that MgSO_4_ can react with ^●^OH preventing the oxidation of cell membranes by stabilization of the radical spin density. However, the possible mechanism of how MgSO_4_ restores or avoid important changes in the properties of oxidized cell membranes is discussed in this work by using MD, by considering a model of the oxidized lipid bilayer of POPC with hydroperoxides (PCHP) and aldehydes (PCAL) as oxidized species, and a low MgSO_4_ concentration. The most relevant issues for the effects of a low magnesium sulfate concentration at different percentages of oxidized species are presented as follows.

(a) Oxidized membrane property changes obtained in this work are in agreement with other theoretical calculations in the literature: a decrease of the thickness (D_HH_), an increase of the area per lipid (A_L_) and, in general, a decrease of the compressibility modulus (K_C_).
(b) The property changes depend on the type of oxidized group; i. e., changes in PCAL are greater than in PCHP. A particular case is PCHP, at low oxidation concentration, where an increase of K_C_ was found, which is in agreement with experimental Results of a decrease in the lateral diffusion.
(c) Results of DD for oxidized species for the phosphate group show the oxidized groups are close to the phosphate groups, in the region of the ester groups. Also, these oxidized groups can be submerged into the other layer and the water region, as in the case of PCAL. This is corroborated with snapshots at the end of the simulation, time evolution position along the Z-direction, and also by the DD for oxidized groups.
(d) There is an important effect of MgSO_4_ to reduce property changes (ΔD_HH_, ΔA_L_, and ΔK_C_) at a low oxidized percentage (<12.5%) for PCHP and at higher values (25%) for PCAL. However, the effect is reversed, concerning the oxidized bilayer without MgSO_4_, at the highest oxidized concentration (50%).
(e) Results of DD showed that MgSO_4_ reduces the spread of the density distribution for oxidized species in the Z-axis at different oxidation concentrations, as compared with the system without the salt. This is an indication the most important effect is in the decrease of oxidized species mobility, particularly the aldehydes in the direction toward the membrane surface
(f) A long- and short-range electrostatic interactions between the Mg^+2^ ion and oxidized species is confirmed by well-defined peaks in the radial distribution function (RDF), as it has been proposed in the literature by experimental Results. This reduces the movement of polar oxidized groups toward the surface interface. The electrostatic interaction between the Mg^+2^ and SO_4_^-2^ ions with oxidized groups is also reflected in the changes of the DD with the membrane oxidation. These results suggest that there is a screening of the surface negative and positive charges by Mg^+2^ and SO_4_^-2^ ions, respectively. The interaction of Mg^+2^ and SO_4_^-2^ ions with phosphate and choline groups, respectively, also stabilizes the surface structure, decreasing the possibility of pore formation.
(g) It would be interesting to perform further studies by changing MgSO_4_ concentrations at different percentages of membrane oxidation. Also, it important to contemplate a mixture of several oxidized species as a model of oxidized membranes.
(h) It is noteworthy to say that this work considers in vitro systems since in vivo other effects should be taken into consideration, such as the known important role of Mg^+2^ in the synthesis of cell antioxidants and its role in the formation of repairing enzymes for oxidized membranes.

## Disclosures

None.

## Declaration of competing interest

The authors declare that they have no known competing financial interests or personal relationships that could have appeared to influence the work reported in this paper.
